# A Systematic Review of Randomized Controlled Interventions for Parents' Distress in Pediatric Leukemia

**DOI:** 10.5402/2011/959247

**Published:** 2011-08-25

**Authors:** A. Bougea, C. Darviri, E. C. Alexopoulos

**Affiliations:** Stress Management and Health Promotion Postgraduate Course, Medical School, University of Athens, 115 27 Athens, Greece

## Abstract

*Objective*. This review aims to summarize the existing evidence concerning interventions towards reducing stress in parents with a child with leukemia and their effect in child and family wellbeing. *Methods*. A systematic review strategy was conducted using MEDLINE covering the period January 1980 to June 2010. *Results*. Seven randomized controlled trials met the inclusion criteria including in total 1045 parents participants. A variety of cognitive-behavioral interventions problem-solving skills training programs have been used for managing distress in parents and children. Outcome measures are assessed by self-report, observer report, behavioral/psychological, and physiological measures. The most prominent methodological problems were the marked heterogeneity in stress measurement and the relative absence of proper measurement and adjustment of moderating and mediating factors. The largest effect has been obtained by combined cognitive-behavioral interventions with promising but limited evidence for several other psychological interventions. *Conclusions*. Recommendations for future RCTs are provided, and particular attention to the quality of trial design and reporting is highlighted.

## 1. Introduction

One third of cancer cases reported in children are leukemia cases [1]. Nowadays, the acute lymphoblastic leukemia, the most common type of childhood leukemia, has a very high rate (60–70%) of survival [2]. However, parents of children with leukemia experience a variety of negative emotions such as shock, disbelief, fear, hopelessness, anger, guilty, and loss of control following diagnosis [3]. In addition high levels of parental psychological distress remain throughout their child's treatment or long after cessation of treatment [4, 5].

A cascade of negative effects of this psychological distress is manifested in parental posttraumatic stress syndrome (PTSS) [6–11] depression, somatization disorders [12], economic burdens [13], and family tensions [14]. Furthermore, in several studies the results indicated that siblings of children with cancer are at risk for emotional, internalizing problems and/or behavioral, externalizing problems [15–21].

Recognizing the necessity of managing parental distress, research has focused on evaluating the effect of psychological and other interventions [22, 23]. Findings regarding intervention effects on specific psychological outcomes have been inconclusive [3]. The diversity of settings, of interventions' targets and content, and of measurement tools makes difficult any comparison and assessment of intervention efficacy. Furthermore little research has been conducted on interventions in which parents are trained to be primarily interventionists [24].

Given the absence of a comprehensive review of interventions for parental distress in pediatric leukemia, we undertook a review in order to summarize the existing evidence concerning the following hypotheses: (a) have interventions shown efficacy in reducing parental stress, (b) what are the effects concerning the children's and the family wellbeing, and (c) what are the gaps or methodological differences between studies.

## 2. Methods

### 2.1. Search Strategy

We identified relevant studies by searching randomized controlled trials (RCTs) published in MEDLINE from 1980 until June 2010. English language restrictions were applied. Search terms were “leukaemia” and “stress” combined with “leukaemia” and “child” or “parent” that limited the search in “randomized control trial(s)” or “intervention”. We carefully selected publications by titles and abstracts referring to our aim and then we tried to get full access on all the relevant studies. We decided on study eligibility according to recommendations from the Cochrane Handbook for Systematic Reviews of Interventions by including original publications of randomized controlled trials (RCTs). We excluded secondary data analyses, case reports, case series, and RCTs that did not report patient outcomes. We have also looked CINAHL and Cochrane databases and the reference lists from systematic reviews but we have not identified further studies. 

Finally, we proceeded by extracting information pertaining to the following characteristics: randomization scheme, intervention characteristics (type, duration, and procedure), stress measurement, number, age, and gender of participants, duration of disease, auxiliary factors measured, and main results. Study quality was analyzed by using the following criteria: participant selection, length and loss of follow-up, masking of the effect evaluation, adequacy of randomization and allocation concealment, and justification of sample sizes. The main characteristics and main results with critical comments are presented in tables and main text.

## 3. Results

Finally out of thirty-four potentially relevant identified studies seven met our selection criteria. Reasons for exclusion were lack of randomisation and/or blinding, not reported patient outcomes, or the study population or the intervention of interest. The studies included in the review are summarized in Table 1.

The first report of Kazak et al. [22] was a randomized, controlled, prospective study based on the Analgesia Protocol for Procedures in Oncology (APPO). The treatment in this study of 286 parents compared a pharmacologic-only protocol (PO group) to the same pharmacologic protocol plus a preventive, parent-centered psychologic intervention (CI group). There was a control group of patients with leukemia in first remission prior to the initiation of APPO as a program for procedural pain and the major dependent variables were child and parent distress based on parent and staff ratings. The groups of this prospective study, with random assignment, were stratified by age, to either group at diagnosis of leukemia in a child under age 18 and self-report scales. Patients were accrued for 36 months. Prospective data were collected at 1, 2, and 6 months after children's diagnosis.

In Walker et al. study [25], 32 couples with a chronically ill child, including leukemia, seen at a tertiary care pediatric hospital were block randomized to either the intervention group (16 couples) that received 10- up to 90-minute Emotionally Focused Therapy sessions every 1- to 2-weeks or to a wait-list control group (16 couples). The couples had been married on average for 9.8 years and averaged 2.2 children. The types of illnesses they were dealing with included cancer, cystic fibrosis, spina bifida, and autoimmune disorders. Eighty-one percent (*n* = 13) of treatment couples participated in the 2-year follow-up. No treatment couple reported receiving psychological intervention for either themselves or their child between the end of treatment and the 2-year follow-up. 

The study by Hoekstra-Weebers et al. [3] conducted in the University Hospital of Groningen included 30 couples of parents and one widow randomly assigned to the intervention group and 28 couples and 3 mothers as the control group. The medical diagnoses included mainly leukemias (*n* = 17) but also malignant lymphomas (7), Wilm's tumor (5), brain tumors (4), soft tissue sarcomas (4), and others (4).

Sahler et al. [23] assessed 430 English and Spanish mothers most of them with a high educational level by a two-arm randomized clinical trial of usual psychological care (UPC) as the control condition versus UPC_PSST problem solving therapy as the intervention. 

Kazak et al. [27] had nineteen families, representing 38 parents/caregivers (20 females/18 males) participated. Nine families were randomized to the treatment arm and ten to the control group. There were no significant differences in demographic variables between the two groups. The treatment arm consisted of three 45-minute sessions of a manualized family intervention for parents/caregivers of newly diagnosed pediatric oncology patients, SCCIP-ND. The treatment sessions conducted within 4–6 weeks after diagnosis focus on identifying the caregivers' beliefs about the adversities associated with cancer and reframing these beliefs to alter unwanted consequences (e.g., distress, relationship difficulties).

In Stehl et al. [28] study Thirty-eight families were randomized to the SCCIP-ND group and 38 to the control group for Standard Psychosocial Care. Eligible families were English-speaking and had a child between birth and 17 years of age who was receiving chemotherapy and/or radiation treatment at hospital, had no medical comorbidities or developmental delay, and was not referred to palliative care.

The included studies assessed a psychoeducational intervention program for parents of pediatric cancer patients, using cognitive and behavioral techniques [3]. The manual-guided intervention consisted of eight 90-minute sessions, during the first six months following diagnosis (a three-week interval between sessions), and involved parent(s) and the psychologist, the outcome data comparing the pharmacologic and the combined intervention conditions to families [22], and the efficacy of problem-solving skills training (PSST), a cognitive-behavioral intervention based on problem-solving therapy on mothers with recently diagnosed children [23]. 

The study of Kazak et al. [22] as a more complex study compared the outcome of a pharmacologic only (PO) and a combined intervention (CI) and showed that mothers in the CI group perceived a lower level of their child's distress during the procedure than mothers in the PO group. The nurses' ratings supported this finding. However, the majority of measures, including mother and father report, and staff ratings of parent and child distress, showed no significant effects of the condition over the PO condition. Parents' perceptions of procedural distress assessed by the Perception of Procedures Questionnaire (PPQ) showed significant differences between the groups indicating that pharmacologic intervention alone as well as the combined intervention condition was associated with low to moderate levels of distress. 

In Walker et al. 1996 [25] study the Emotional Focused Therapy—a type of therapy which focuses on building a secure bond between spouses—showed to be effective in treating general marital distress. There was a significant main effect of time on Dyadic Adjustment Scale DAS and a significant difference between pretreatment and posttreatment scores on the DAS. Moreover, there was no difference between DAS scores at posttreatment and 2-year follow-up indicating that maintenance was achieved. No significant time effect was found on the Miller Social Intimacy Scale MSIS. It should be noted that there were differences in DAS scores between partners at pretreatment, posttreatment, and follow-up with the most pronounced differences and variability across couples seen at follow-up. There were no statistically significant correlations between the number of life stressors identified by parents as measured by the Parent Stress Index, Life Stress Scale, and marital satisfaction or intimacy as measured by the DAS and the MSIS at pretreatment, posttreatment, or 2-year follow-up. 

In the study of Hoekstra et al. [3] after a psychoeducational manual-guided program the study group (control and intervention parents, *n *= 81) was compared to the parents (*n = *39) who dropped out of the trial and there were no differences between these two groups on the demographic variables and on the outcome variables (psychological functioning, social support, and intensity of emotions), at baseline. Since contact with a social worker was at the initiative of the parents, there may have been differences between parents in the two conditions in the number of meetings. No differences were found. Although there was a decrease in parental distress over time, there was no significant decrease in distress between the intervention and the control group in psychological distress (GHQ), psychiatric complaints (SCL), state anxiety, negative and positive emotions, and dissatisfaction with support. The authors reported the effect size for the intervention as medium.

The multisite randomised trial of Sahler et al. [23] used a Problem-Solving Skills Training (PSST) programme and a cognitive behavioural intervention to mothers of recently diagnosed children. Administering PSST resulted in significant decrease both in maternal negative affectivity and overall in differences between the groups immediately following the intervention in the Social Problem-Solving Inventory Revised (SPSI-R) summary score and in the subscores for negative problem orientation (NPO) and avoidance style (AS). There were also statistically significant differences in all of the measures of negative affect, the measures of Profile of Mood States, Beck Depression Inventory II, and Impact of Event Scale Revised (POMS, BDI-II, and IES-R). Although differences were maintained at 6 months (3 months after the intervention) for the BDI-II and IES-R measures, other differences between the UPC (usual psychological care) and PSST and UPC groups diminished over time, primarily attributable to slow but continued improvement in the control mothers.

Another study by Kazak et al. [27] used the Surviving Cancer Competently Intervention Program Newly Diagnosed, SCCIP-ND. Outcome data showed changes in the desired direction like reduced anxiety and parental posttraumatic stress symptoms (PTSSs). 

Stehl et al. [28] also focused on the feasibility and outcomes from the Surviving Cancer Competently Intervention Program for Newly Diagnosed Families (SCCIP-ND) where no significant differences were observed in Acute Stress Disorder Scale (ASDS) scores at Time 1 (T1) data collection between the SCCIP-ND and Control groups and at Time 2 (T2) data collection 1 month following the third session for families in SCCIP-ND in the Impact of Event Scale-Revised (IES-R) for primary caregivers or secondary caregivers. On average, both SCCIP-ND and TAU groups showed a significant decrease of state anxiety from T1 to T2 for primary and secondary caregivers. When analyzing primary and secondary caregivers separately, the data indicated no differences in STAI scores at T2 between groups for primary caregivers or secondary caregivers.

The levels of stress in parents of children undergoing bone marrow transplantation and how a psychological intervention program manages the stress were studied by Streisand et al. [26] Based on a stress inoculation model one 90-minute intervention session focused on education, relaxation, and education. In addition, parents were provided handouts and a tape of relaxation techniques. Mothers in the intervention condition reported use of a greater number of intervention techniques than others in the standard care condition. Mothers in the intervention group reported less stress on the Daily Stress Inventory (DSI) and Parenting Stress Index (PSI) both prior to and 21 days post transplant. On the Semistructured Interview (SSINT), there was no significant difference between the control and intervention group. The effect size for both the DSI and PSI was medium to large. 

This stage of the review aimed to critically summarize the evidence about the effectiveness of specific psychological intervention strategies in improving the outcomes of cancer patients. Unfortunately, a number of limitations within the trials themselves hampered our ability to make strong recommendations about any of the intervention strategies. The most important ones were that in the work of Kazak et al. 1996 [22] eligibility criteria were not specified. In the same study and also the work of Walker et al. 1996 [25] randomisation was not adequately reported, and also in most studies (except Streisand et al. [26]), allocation consealment was not adequately reported.

## 4. Discussion

We identified a growing body of literature that explored the effectiveness of psychological therapies and distress interventions for cancer patients and their parents. Despite the increased use of randomized, controlled trial designs over time, the methodological quality of most of the trials that we reviewed was not optimal. Many of the trials failed to provide sufficient information in order to assess their performance on many of the methodological indicators. 

As far as it concerns the effectiveness, the study of Kazak et al. [22] showed that the participated subjects under a combined intervention had lower child's distress than the other group subjects under pharmacological intervention. The study of Walker et al. [25] showed a significant main effect of time on Dyadic Adjustment Scale DAS and a significant difference between pretreatment and posttreatment scores on the DAS, and Hoekstra-Weebers et al. [3] noticed an effective result concerning the less experienced distress of parents who followed a psychoeducational manual program. Sahler et al. [23] have shown positive effects because the PSST resulted in significant decrease both in maternal negative affectivity and overall in differences between the groups immediately following the intervention in the Social Problem-Solving Inventory Revised. In Streisand et al. [26] study after repeated statistical measures there were noticed significant changes in mother's stress and the same effectiveness was shown by Kazak et al. [27] using the Surviving Cancer Competenly Intervention Program Newly Diagnosed with which they achived reduced anxiety and parental posttraumatic stress. At last Stehl et al. [28] focusing on a Surviving Cancer Competently Intervention Program for Newly Diagnosed Families (SCCIP-ND) showed no significant impact in measures of state anxiety and posttraumatic stress symptoms. 

It has to be mentioned that other studies—not fulfilled our eligibility criteria—have shown promising results. These included the study by Field et al. [24], that assessed the effects of massage therapy on anxiety, depressed mood, immune function in children, and also how this technique benefits the elderly and the results suggested that the massage therapy group parents had lower anxiety and depressed mood levels after the massage therapy sessions on the first day of the study. The study by Hashemi and Shokrpour [29] has shown a possible moderating effect of parents' knowledge and education level on the needs of the pediatric cancer patient and the study by Dragone et al. [30] has shown a positive effect of a CD-ROM because it was found to be a useful, engaging, and empowering tool for children by increasing the feeling of control.

Several limitations were identified in the specific studies, mainly by their authors, limiting their external validity. In Kazak et al. 1996 [22] study, mothers' perceptions biased by their very involvement and the PSI-S may be less sensitive to the procedural context. In Walker et al. 1996 [25] study, as part of the study design, the therapists were given specific training on how to deal with parents of chronically ill children and were also given medical information specific to the diseases in question, which as a confounding variable might had been responsible for the study's positive results, as opposed to the emotional focus of the therapy. In the study by Hoekstra-Weebers et al. [3] the intervention may not have addressed the specific problems of the parents, being too general, and had insufficient time and the questionnaires were generic and insensitive. In Streisand et al. [26] study, the sample was small and a selection bias is possible. The study of Kazak et al. 2005 [27] had lack of information about intervention and measurement (use and measurement of the Bright IDEAS problem-solving strategy) and inadequate time frame. In Stehl et al. 2009 [28] study there were difficulties enrolling participants that resulted in a low participation rate and also a relatively short follow-up period. In all studies some observer (non blindness) bias is possible and putative moderating, mediating, or confounding factors were not taken into account. 

Additionally, there are some limitations in our study that we should consider. One limitation of this review is that we searched a limited number of databases. Thus, there is always the possibility that we have missed studies in other databases and published before 1980. In addition, there could always be problems such as publication bias due to under-report of negative results and grey literature. However, we used little search limits increasing the sensitivity of search method. Finally, 3 out of 25 studies were retrieved in abstract forms limiting our ability to evaluate systematically. 

## 5. Overview and Future Directions

The variety of interventions did not allow any synthesis of the results on effectiveness. However, different approaches like group therapy, education, structured, and unstructured counselling, and cognitive behavioural therapy appeared to provide potential benefits both in medium- and long-term basis in most of the psychosocial outcomes studied. The comparative lack of immediate- and short-term benefits could suggest that psychological therapies are more likely to offer psychosocial benefits over the longer term. This finding, however, may well be biased by the small number of trials that assessed the long-term effects of intervention strategies. Most studies did not take into account possible moderators or mediators and such factors, if ignored, could easily over or underestimate the results. In addition, most studies do not measure or adjust for other triggering factors. Future studies should incorporate such measures.

## Figures and Tables

**Table 1:** Randomized interventions towards reducing stress in parents with a child with leukemia.

Study	N parents	Mean age	Gender	Education level	Type of intervention	PsyMesures
Kazak et al. 1996 [22]	C: 134 I: 152	Not available	F:157 M:129	Middle	PO group: pharmacologic only according APPO protocol. CI group: pharmacologic protocol + preventive, parent-centered psychological intervention	PPQ
						PSI-S
						Parent & staff ratings of distress

Walker et al. 1996 [25]	C: 32 I: 32	F: 35 M: 37,7	Not reported	Not reported	C group: Standard care. I group: Emotionally Focused Therapy	DAS
						MSIS
						PSI

Hoekstra-Weeberset al. 1998 [3]	C: 42 I: 39	36.6	F: 41 M: 40	Elementary to university	C group: routine medical and psychosocial care I group: Eight, 90-minute manual-guided interventions based on Psychoeducational, cognitive-behavioural techniques	GHQ
						SCL
						Stais
						Dissup
						Emoneg
						Emopos

Streisand et al. 2000 [26]	C: 11 I: 11	C: 36.5 I: 37.2	F: 22	Middle to high	C group: Standard care preparation I group: Stress inoculation model	DSI
						PSI
						SSINT

Kazak et al. 2005 [27]	C: 20 I: 18	Caregivers: 35 Control: 37	F: 20 M: 18	C: low to advanced I: medium to advanced	C group: Usual psychosocial care I group: Surviving Cancer Competently Intervention Program Newly Diagnosed	ASDS
						IES-R
						STAI


Sahler et al. 2005 [23]	C: 213 I: 217	Not reported	F: 430	High	C group: Usual psychological care I group: Usual psychological care and problem-solving skills training (PSST) programme	NEO-FFI
						SPSI-R
						POMS
						BDI-II

Stehl et al. 2009 [28]	C: 62 I: 62	35	Not reported	<12th grade to College/advanced degree	C group: Standard psychosocial care I group: Surviving Cancer Competently Intervention Program Newly Diagnosed	ASDS
						IES-R
						STAI


C: control group; I: intervention group; F: females; M: males; PPQ: Perception of Procedures Questionnaire; PSI-S: Parenting Stress Index-Short Form; GHQ: General Health Questionnaire; SCL: Symptom Check List; Stais: state anxiety; Dissup: Dissatisfaction with support; Emoneg: negative emotions; Emopos: positive emotions; DSI: Daily Stress Inventory; PSI: Parenting Stress Index; SSINT: Semi-structured Interview; ASDS: Acute Stress Disorder Scale; IES-R: Impact of Event Scale-Revised; NEO-FFI: NEO-Five Factor Inventory; SPSI-R: Social Problem-Solving Inventory-Revised; POMS: Profile of Moods States; BDI-II: Beck Depression Inventory-II DAS: Dyadic Adjustment Scale; MSIS: Miller Social Intimacy Scale.
